# A community-based, medical student-led walking and education program was associated with a reduction in frailty levels among adults with elevated frailty

**DOI:** 10.3389/fragi.2025.1690493

**Published:** 2025-11-25

**Authors:** Myles W. O’Brien, Taylor M. Wilson, Muhammad Cheema, Zainab Zafar, Minji Choi, Madeline E. Shivgulam, Olga Theou

**Affiliations:** 1 Department of Medicine, Université de Sherbrooke, Sherbrooke, QC, Canada; 2 Centre de Formation Médicale du Nouveau-Brunswick, Université de Sherbrooke & Université de Moncton, Moncton, NB, Canada; 3 Department of Medicine, Dalhousie Medicine New Brunswick Campus, Dalhousie University, Saint John, NB, Canada; 4 Geriatric Medicine Research, Dalhousie University and Nova Scotia Health, Halifax, NS, Canada; 5 School of Physiotherapy (Faculty of Health) and Division of Geriatric Medicine (Faculty of Medicine), Dalhousie University, Halifax, NS, Canada

**Keywords:** physiological aging, community exercise program, health education, physical activity, Walk with a Doc, student engagement

## Abstract

**Objectives:**

Frailty reflects the accumulation of health deficits that an individual develops over their lifespan. Walk with a Future Doc (WWAFD) is a medical student-led, community-based education and walking program. We examine the impact of a 12-week WWAFD program on lowering the frailty levels in a New Brunswick community and if the effects of the program would be specific to those with higher pre-WWAFD frailty levels.

**Methods:**

Eighty participants (age: 41–85 years; 51 female individuals) were recruited from the YMCA in New Brunswick (Canada) via word-of-mouth, social media, and consulting local physicians. The inclusion criteria were broad. All community members were welcome to attend the program, but only those over age 18 and those that attended ≥6/12 walks were included in the study. Participants were grouped into non-frail (FI< 0.10; n = 51) and very mild + frail (FI ≥ 0.10; n = 29) groups for comparison. Participants attended a student-led 1-h/week health education and walking program and completed the Canadian Longitudinal Study on Aging Frailty Index (FI) questionnaire before and after the program.

**Results:**

Frailty was unchanged pre-to-post-intervention in the non-frail group (0.053 ± 0.030 to 0.056 ± 0.029, *p* = 0.475) but reduced in the very mild + frail group (0.166 ± 0.075 to 0.153 ± 0.066, *p* = 0.014).

**Conclusion and implications:**

The WWAFD program that included weekly walking and education sessions was associated with reduced frailty levels among adults with FI ≥0.10. This change emphasizes the value of community-based physical activity programs and exemplifies the impact they can have on the participants’ health outcomes.

## Introduction

Frailty, commonly operationalized in healthcare and research as the accumulation of health deficits, accounts for the heterogeneity in aging and provides insight into why two individuals of the same age may exhibit divergent health statuses (Xue, 2011). Adults with higher frailty levels are more susceptible to the insults of minor illnesses and are at an increased risk for additional chronic conditions, hospitalization, and all-cause mortality (Xue, 2011; Kojima et al., 2019; Pérez-Zepeda et al., 2021). Although the positive effects of physical activity on frailty are established based on exercise-training studies (Aguirre and Villareal, 2015), programs that focus on middle-aged and older adults in the community that include simple, easy-to-do, low-resource activities are less common. It is well-recognized that walking is the most accessible form of physical activity for middle-aged and older adults (Liu and Fielding, 2011). Walk with a Doc is a not-for-profit international organization that aims to increase the community levels of health education, physical activity, social connectedness, and time spent outdoors (Sabgir and Dorn, 2020). Subsets of the program also exist across the globe, one of which is the Walk with a Future Doc (WWAFD) program. While primarily resource-intensive, highly-structured laboratory-based exercise training studies have demonstrated positive effects on frailty (Theou et al., 2011; Shivgulam et al., 2025a), community-based interventions may serve a unique role in reaching more people and might be more sustainable models of health promotion. WWAFD is unique in that it is free, community-driven, and led by medical students, thereby encouraging early collaboration and connection amongst healthcare trainees with patients (Wilson et al., 2024). Although the Walk with a Doc and WWAFD models are designed to help community members, the effectiveness of the medical student-led WWAFD program to improve health outcomes, such as frailty, is not well-characterized. Understanding the impact of the WWAFD program on the frailty levels could position this unique model of exercise and education as a useful tool for regions to develop similar programs in efforts to reduce the development of health deficits.

Approximately 25% of middle-aged (45–64 years) and older adults (≥65 years) exhibit frailty levels at a very mild or higher level (i.e., frailty index >0.1 or >10% of deficits measured; clinical frailty scale of ∼4), whereas 75% are non-frail (frailty index <0.1) (Pérez-Zepeda et al., 2021). Although adults with higher frailty may benefit the most from leading a physically active lifestyle, they may have the greatest challenges performing physical activity (Kehler et al., 2020). Conversely, those who are non-frail (e.g., <0.1) are unlikely to exhibit changes in frailty from physical activity programs following the typical timeline of movement interventions (e.g., 8–12 weeks) (Kehler et al., 2020). We sought to examine the impact of pre-WWAFD frailty levels on the potential changes in frailty following the WWAFD program. We test the hypothesis that the WWAFD program will lower the frailty levels of adults with higher pre-WWAFD frailty levels, but those with lower pre-WWAFD frailty levels will remain unchanged.

## Methods

### Participants

Members of the greater Saint John (New Brunswick, Canada) area were invited to attend free weekly walks at a local YMCA facility. Local primary care providers interested in supporting the program were given posters and prescription pads to encourage their patients to attend. Medical trainees encouraged primary care providers to refer their patients to the program and advertised the program on social media and via word-of-mouth.

WWAFD was initially implemented by medical students as a community physical activity and education program. A secondary research component was led by medical students that aimed to answer our proposed research question. All participants were welcome, regardless of age. There were no mobility or baseline health condition criteria for participants to be included in the program. Our target population was those 40 years of age or older due to frailty emerging in middle-aged years (Pérez-Zepeda et al., 2021), and data were not collected from those under 18 years of age (n = 1). To answer our proposed research question, a sample size calculation using a repeated-measures ANOVA model indicated that a minimum of eight participants per group was needed for a within-between interaction, based on a moderate effect size (f = 0.25), β = 80%, α = 0.05, and a 0.80 correlation between repeated measures. All protocols and procedures conformed to the Declaration of Helsinki and were approved by the Human Health Research Protection Program and the Research Ethics Board at Horizon Health Network (#101609). Written and informed consent was obtained from all the participants prior to data collection.

### Study design

Participant demographics (i.e., age, sex, gender, and chronic health condition history) were collected before the walk via the Canadian Longitudinal Study on Aging Frailty Index (CLSA-FI) questionnaire. At the end of 12 weeks, participants were asked to complete the CLSA-FI again. To answer the proposed research question, those who attended ≥6/12 sessions (n = 80/118 participants) were included in the analyses. A 50% attendance rate was selected as a heuristic threshold to alleviate concerns that a lack of change in CLSA-FI is due to poor attendance.

### Walk with a Future Doc program

The WWAFD program and favorable perceptions of the program from the attendees are described in detail elsewhere (Wilson et al., 2024; Shivgulam et al., 2025b). The 12-week program included one 60-min session/week, consisting of a 10-min health education talk given by a medical student followed by a 50-min self-paced walk on an indoor track. The average walking distance covered by each participant was approximately 3.5 ± 0.8 km/session. The health education talks that were given included a variety of topics: diet and nutrition, sleep hygiene, chronic pain management, spending time in nature, Canada’s 24-h movement guidelines, stretching, balance, staying motivated to exercise, setting SMART goals, Canada’s low-risk alcohol drinking guidelines, hydration, and the impacts of social interaction on mental health. The program was led by medical students, whose role was to promote the program, undertake research data collection, provide health talks, and walk with the participants. At least one physician was mandated to be in attendance at each walk. In addition to allowing the participants to engage with healthcare providers and medical students, the walk also provided a space for participants to socialize with other community members. The ratio of community members to medical trainees was approximately 5:1, and there were ∼1–3 physicians at each walk.

### Frailty index

The frailty index implemented in the present analysis was based on the deficit accumulation model (Mitnitski et al., 2001) and was initially developed and validated against mortality records using the CLSA dataset (Blodgett et al., 2022), following standard procedures (CLSA-FI) (Searle et al., 2008). The 65 questions (Supplementary Material S1) are compiled from five domains within the questionnaire: activities of daily living (12 questions), mobility (13 questions), self-rated health (4 questions), previous falls and effort (4 questions), and chronic health conditions (32 questions). The individual items included were coded as 0 (no deficit) or 1 (deficit). Interval or ordinal variables were coded as a proportion of complete deficit (e.g., self-rated health has five options: excellent = 0, very good = 0.25, good = 0.5, fair = 0.75, and poor = 1). As a 65-item questionnaire, the CLSA-FI well-exceeds the required 30 deficits needed to reliably calculate a frailty index (Searle et al., 2008). The overall frailty index was calculated as the deficit score ÷ the number of deficits measured for each participant (e.g., 6.5/65 = 0.10), with a value closer to 1.00 indicating a higher degree of frailty. Based on the available data and established thresholds, participants were characterized as non-frail (CLSA-FI<0.1) or living with very mild + frailty (CLSA-FI: ≥0.10) (Blodgett et al., 2022). The rate of missing responses to the questionnaire was low (i.e., all participants answered >64/65 questions).

### Statistical analyses

All dependent variables were assessed for normality using a Shapiro–Wilk test and homoscedasticity via visual inspection and determined to be normally distributed (all Shapiro–Wilk, *p* > 0.10). Independent sample *t*-tests or chi-squared tests were used to compare the participant characteristics between groups. We conducted a two-way, group (non-frail: FI < 0.10 vs. very mild + frail: FI ≥ 0.10) by time (pre-WWAFD and post-WWAFD) repeated-measures analysis of covariance with Bonferroni *post-hoc* testing. Age and sex were included as covariates. The variance of differences was assessed using Mauchly’s test of sphericity, and the Greenhouse–Geisser correction factor was applied to the degrees of freedom used when assumptions of sphericity were violated. Within-group effect sizes are reported as Cohen’s *d*. All statistical analyses were completed in IBM SPSS Statistics version 29 (IBM Corp., Armonk, NY, United States) with a statistical significance threshold of α = 0.05. Data were presented as the mean ± standard deviation.

## Results

Eighty participants were included in the present study. Participants were 65 ± 8 (range: 41–88; 40 participants ≥65) years old, had 3.4 ± 2.9 chronic conditions, and were primarily female (51/80 participants) and Caucasian (79/80). One person self-identified as African Canadian. On average, the very mild + frail group participants were older and had more chronic conditions (both, *p* < 0.018; Table 1), but the sex distribution and proportion of those attending 10–12 walks were not different (both, *p* > 0.222). Prior to starting the program, 51/80 (31 female individuals) were non-frail (FI < 0.10) and 29/80 (20 female individuals) had frailty levels of very mild+ (FI ≥ 0.10). By design, the very mild + frail groups had higher overall frailty scores than the non-frail group (*p* < 0.001). No differences in the number of walks attended between groups were observed (very mild+: 9.6 ± 2.1 vs. non-frail: 9.2 ± 2.1, out of 12; *p* = 0.407; *d* = 0.19).

**TABLE 1:** Participant characteristics based on the pre-program frailty level.

Variable	Non-frail (n = 51)	Very-mild + (n = 29)
Age (years)	63.5 ± 7.8	67.6 ± 6.9*
Sex (# female, male)	35, 16	18, 11
Gender (women, men)	35, 16	18, 11
Race (# Caucasian, African Canadian)	50, 1	29, 0
Attended walks (6–9, 10–12)	27, 24	12, 17
Number of chronic conditions	1.8 ± 1.5	6.3 ± 2.6*
Pre-program frailty level	0.05 ± 0.03	0.16 ± 0.07*

Data presented as the mean ± SD or proportions (%). Data were compared using chi-square or independent samples *t*-tests. *vs Non-Frail.

**FIGURE 1:**
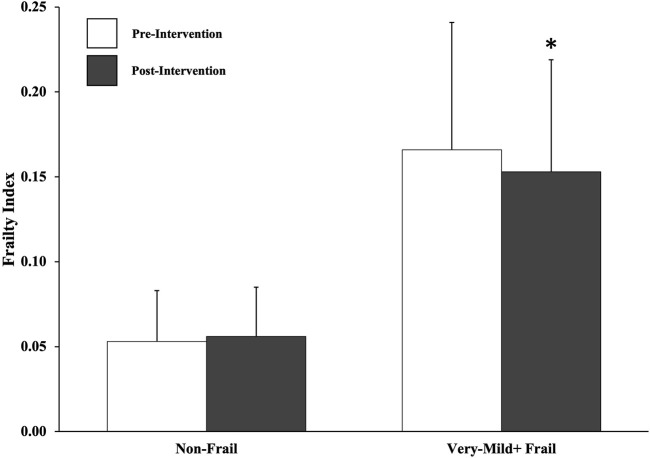
Changes in frailty levels among non-frail (lower frailty index; n = 51) and very mild + frail (higher frailty index; n = 29) participants after 12 weeks of a Walk with a Future Doc program. Frailty scores were determined based on the self-completed Canadian Longitudinal Study on Aging Frailty Index (CLSA-FI) questionnaire. Participants were characterized as non-frail (CLSA-FI<0.10) or living with very mild + frailty (CLSA-FI: ≥0.10) according to the available data and established thresholds. *, *p* < 0.05 compared to very mild + frail pre-intervention. Frailty at both time points were higher in the very mild + group versus the non-frail group (both, *p* < 0.001). Group data are presented as the mean ± SD, and individual participant data are presented as lines.

We observed a group × time interaction (*p* = 0.018) whereby the very mild + frail group was associated with a reduced frailty level from 0.166 ± 0.075 to 0.153 ± 0.066 from baseline to follow-up (*p* = 0.014; Figure 1; *d* = 0.18), but the non-frail group was not (0.053 ± 0.030 to 0.056 ± 0.029, *p* = 0.475; *d* = 0.10). A total of 12 participants (10 from the very mild + frail group) had an FI decrease of ≥0.03. Four (of n = 29) participants with FI ≥ 0.1 pre-program had an FI < 0.1 post-program, thereby shifting their frailty level group following WWAFD completion. The changes in FI (post–pre) for very mild + frail and non-frail groups were −0.013 ± 0.035 and 0.003 ± 0.020, respectively. In exploratory analyses, a group-by-time interaction was observed (*p* = 0.048) when median splitting the participants who attended 6–9/12 walks (*n* = 39; frailty: 0.088 ± 0.078 to 0.091 ± 0.076, *p* = 0.441; *d* = 0.04) versus 10–12/12 walks (*n* = 41; frailty: 0.100 ± 0.071 to 0.091 ± 0.056; *p* = 0.041; *d* = 0.14).

## Discussion

This study implemented a medical student-led community walking program to prevent and manage frailty outcomes in the aging population. We demonstrated that participants with a very mild + frailty level were associated with a better overall frailty score post-WWAFD, but frailty was not associated with a change among those who were non-frail pre-WWAFD.

Higher frailty levels indicate a greater vulnerability to health problems, with long-term (>5 months) structured physical activity generally reducing frailty levels (Theou et al., 2011). Our walking-based community model was associated with favorable improvements in 12 weeks among those with frailty levels ≥0.10 (average pre-WWAFD FI: 0.166 ± 0.075) at baseline. Notably, 4 of our 29 participants in the very mild + frail group started with an overall frailty level ≥0.1, but they had a post-program FI < 0.1. This associated change demonstrates a switch in frailty group after completing the WWAFD program, based on our pre-determined cut-off values, and may help emphasize the power that a community walking program can have on participants’ health. Although based on a hospital setting (Theou et al., 2020) and the minimal clinically important difference is likely lower among community-dwelling older adults, 10/29 of the very mild + frail group exhibited a >0.03 change in FI. The associated improvements in health were likely particularly pronounced among these 10 individuals. Given that the CLSA-FI has been validated against mortality records (Blodgett et al., 2022), the reduction in CLSA-FI observed among the very mild + frail group may be associated with a decreased risk of all-cause mortality. Overall, lowering frailty levels can aid older adults decrease the number and impact of acute illnesses they experience.

Although overall frailty was not associated with an improvement among the non-frail group, the program is plausibly still beneficial for attendees. Given previous observations that the social aspect of WWAFD was a primary reason for attendance (Wilson et al., 2024), we maintain that group-based physical activity of all abilities should be implemented due to the social benefits and, plausibly, the unmeasured potential cardiometabolic effects. The presence of medical students at each walk creates a safe walking environment for the participants, along with a space for members to learn about health and ask health-related questions. Long-term tracking of such participants will provide insights into whether this program prevents the development of frailty over time.

Our study examined the associated frailty changes in a community-based walking program across groups based on their pre-WWAFD frailty levels. However, it lacks a randomized controlled trial design with a non-physical activity control group. Based on the study design, we are unable to discern the impact of regression to the mean, a Hawthorne effect (i.e., frailer persons engaging in additional healthy behaviors due to being monitored), or spontaneous improvement over time (e.g., variations in self-reported mobility). In addition, we acknowledge the potential for confounding variables in that participants in the community voluntarily joined the WWAFD program, likely consisting of healthier participants who were motivated to be physically active. Notably, 80/118 persons attended >50% of sessions, which may contribute to a selection or attrition bias. Presumably, some persons attended single sessions but were uninterested in continuously participating, and this is an aspect that is particularly specific to a free, community-based activity program that is marketed as a health promotion initiative versus a structured training study. Based on prior observations that 25% of Canadian middle-aged and older adults have frailty levels >0.1 and that this is a threshold by which people may be described as living with very mild frailty (versus non-frail), we used this as a threshold for our higher frailty group, but whether these observations would be replicated among those with moderate-to-severe frailty (e.g., >0.30; n = 2 in this study) is unclear.

The generalizability of the results is limited by the fact that our sample consisted predominantly of White female individuals living in a rural community. Additional research is recommended to address these details and gain insight into how participant behaviors differ within (e.g., social interaction with students) and outside of the program, including, but not limited to, their diet, the number of at-home medications, weekly physical activity minutes, and recent immunizations (Rasiah et al., 2022). Strengths of this study include the ease of implementation of a WWAFD program, the free cost for its attendees, and the positive impact it can have on frail adults. The study could be replicated in other communities with an existing Walk with a Doc or WWAFD chapter or as a method of encouragement for other communities to implement similar programs.

## Conclusion

WWAFD was associated with a reduction in frailty levels among middle-aged and older adults with very mild + pre-WWAFD frailty levels, who represent a vulnerable population, after participating in a free 12-week community-based walk and talk program led by medical students. These results signify that community-based physical activity programming is associated with favorable improvements in middle-aged and older adults belonging to this baseline frailty population.

## Data Availability

The raw data supporting the conclusions of this article will be made available by the authors, without undue reservation.
