# Supplementary material for: A community-based, medical student-led walking and education program was associated with a reduction in frailty levels among adults with elevated frailty
Source: Front Aging. 2025 Nov 25;6:1690493. doi: 10.3389/fragi.2025.1690493 (PMC12685861; doi:10.3389/fragi.2025.1690493)
Supplement: Supplementary file 1 [file Table1.docx]

**Supplemental File 1 Canadian Longitudinal Study on Aging Frailty Index (CLSA-FI) Questionnaire.**

Pérez-Zepeda, M. U. *et al.* Frailty among middle-aged and older Canadians: Population norms for the frailty index using the Canadian Longitudinal Study on Aging. *Age Ageing* **50**, 447–456 (2021).

**
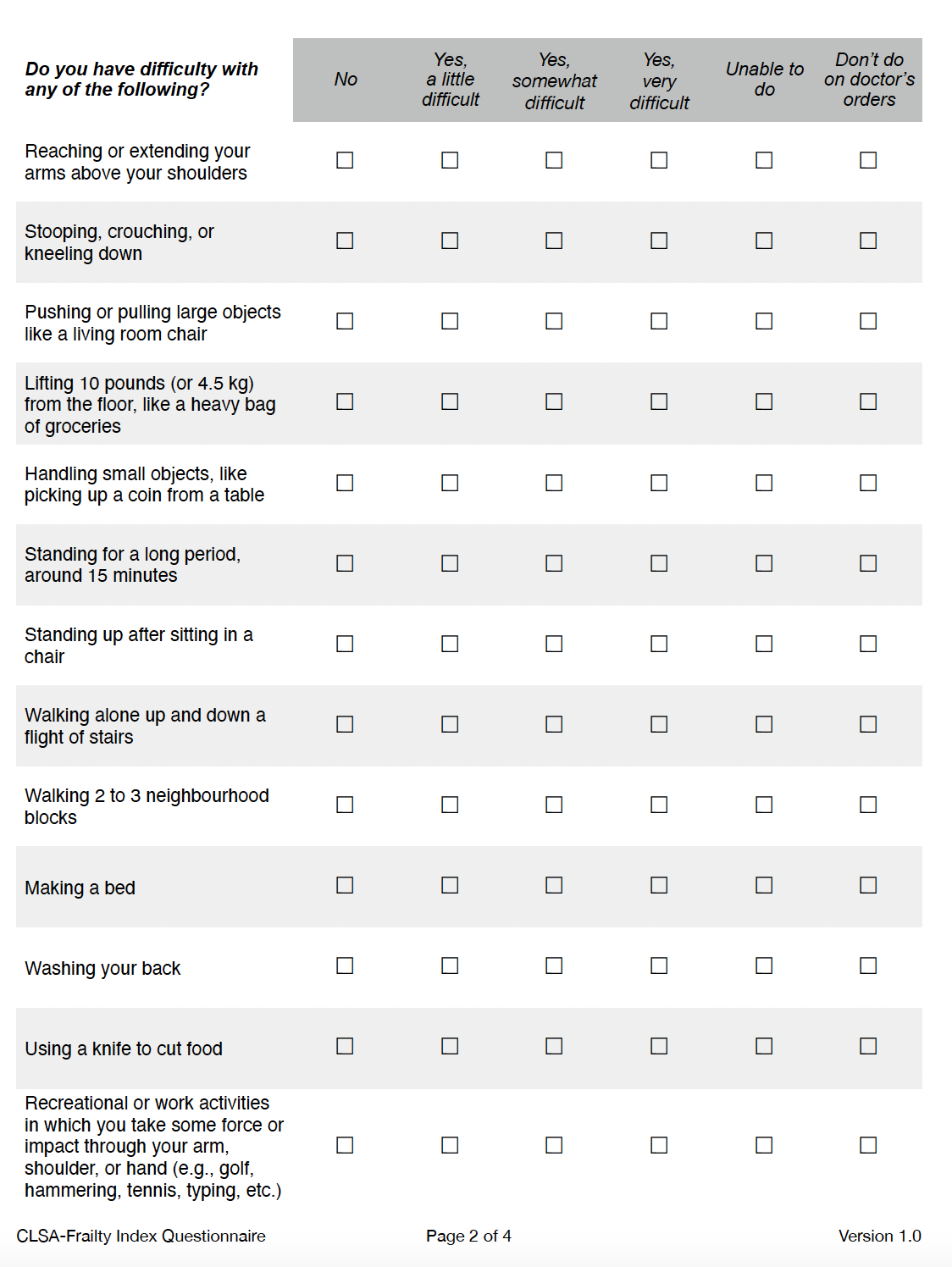
**

**
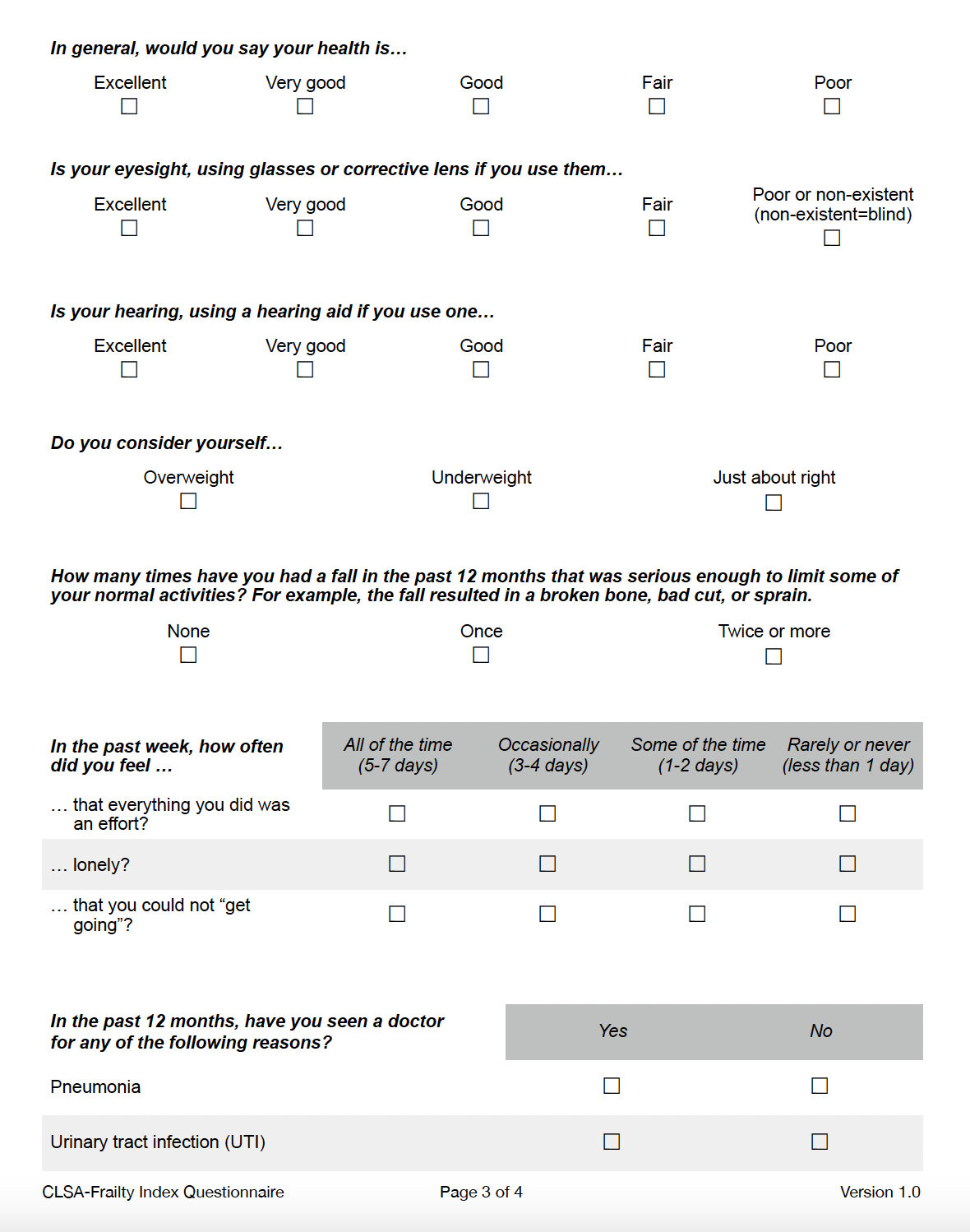
**

**
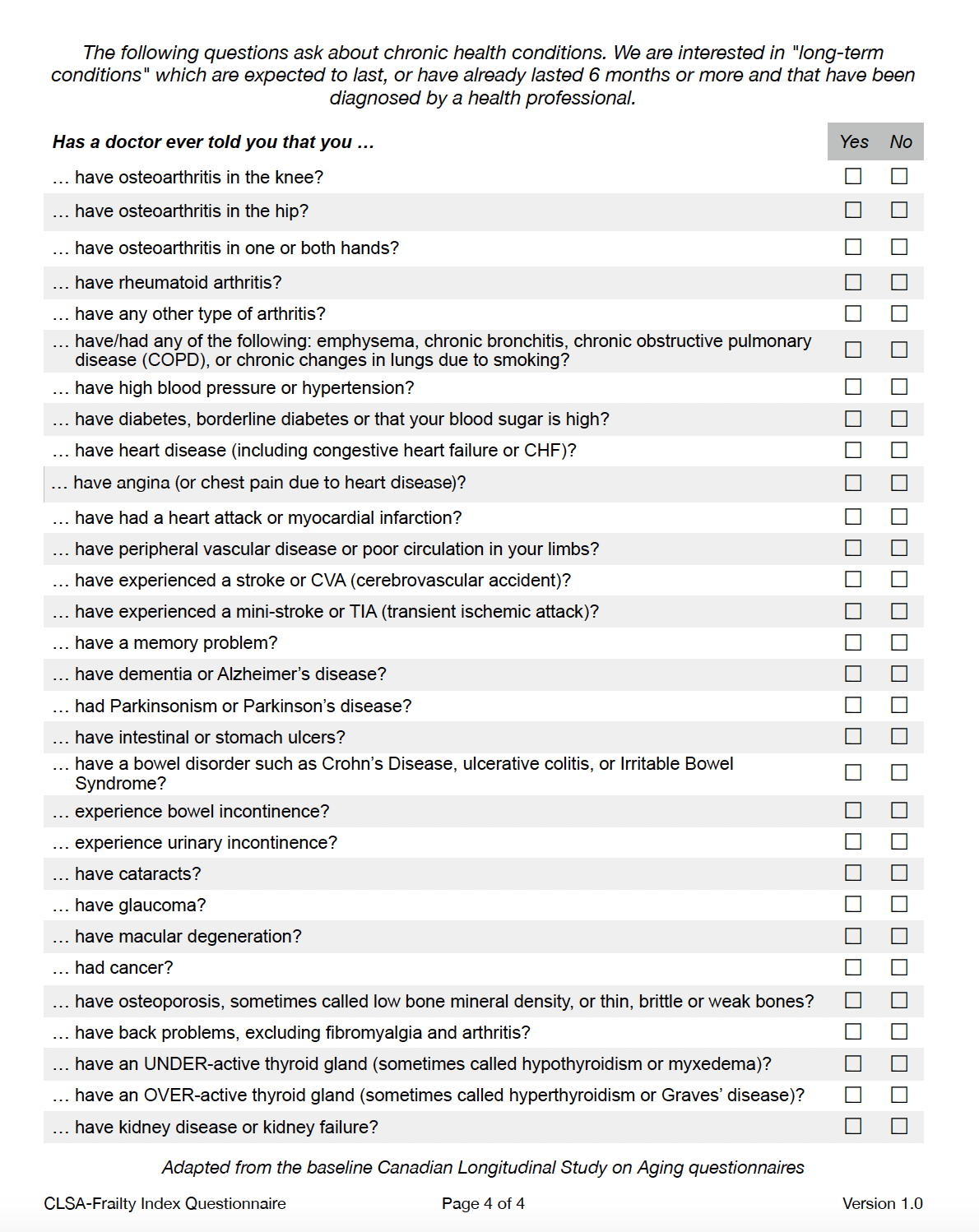
**
